# Evaluations of in-house PCR based diagnostic assay using *pfoB* gene for diagnosis of *Trichomonas vaginalis* among symptomatic women with vaginal discharge

**DOI:** 10.1186/1471-2334-14-S3-E47

**Published:** 2014-05-27

**Authors:** SC Sonkar, PK Mishra, P Mittal, A Kumar, D Saluja

**Affiliations:** 1 Dr. B. R. Ambedkar Center for Biomedical Research, University of Delhi, Delhi-110007, India; 2 Department of Obstetrics & Gynecology, Vardhman Mahavir Medical College and Safdarjung Hospital, New Delhi-110029, India

## Background

*Trichomonas vaginalis* is one of the most prevalent non viral sexually transmitted diseases (STDs) in developing countries. Although several PCR based diagnostic tests are available, the performance, accuracy and sensitivity of these assays vary. Therefore, we decided to compare the performance of various diagnostic gene targets (*18S rRNA*, *β tublin*, *pROS21*) of *T. vaginalis* under similar conditions and also compared an in-house developed PCR assay targeting *pfoB* gene with these methods.

## Methods

Samples were obtained from Department of Obstetrics & Gynecology, Vardhman Mahavir Medical College and Safdarjung Hospital, New Delhi. The DNA so extracted was used as template for PCR amplification using primers targeting *pfoB*, *18S rRNA*, *β tublin* and *pROS21* gene.

## Results

The performance (sensitivity, specificity, positive predictive value and negative predictive value) of different PCR assays was compared using DNA isolated from clinical samples. The *pfoB* PCR showed highest sensitivity (92.31%) and specificity (99.53%) respectively. Our results suggest that though most of PCR based diagnostic assays are highly sensitive and specific, the clinical performance of different primer pairs varies. The positive and negative likelihood ratios were also calculated. The lowest negative likelihood ratio was observed for *pfoB* PCR.

## Conclusion

In the present study we evaluated the diagnostic performance of an in-house developed PCR assay by comparing it with already established PCR methods used for diagnosis of *T. vaginalis*. Therefore, we propose that, it can be used as a reliable diagnostic test for screening *T. vaginalis* infection.

